# 

**Published:** 1889-01

**Authors:** 


					﻿to cts. a copy
JANUARY, 1889.
$i.oo per year.
ft ALLS i
HLAITLI
ESTABLISHED 1854
‘W ar
w.-1.
OFFICE: 206 BROADWAY. EVENING POST BUILDING, Room 11. N. Y.
Entered as Second-class Matter at the Post-Office, New York.
				

